# Spontaneous regression of primary cutaneous diffuse large B‐cell lymphoma, leg type: A case series and review of the literature

**DOI:** 10.1111/1346-8138.17339

**Published:** 2024-07-19

**Authors:** Manuel Winkler, Jana Dorothea Albrecht, Christian Sauer, Theresa Kordaß, Emmanuella Guenova, Elisabeth Livingstone, Marion Wobser, Christina Mitteldorf, Cyrill Géraud, Jan Peter Nicolay

**Affiliations:** ^1^ Department of Dermatology, Venereology, and Allergology, University Medical Center and Medical Faculty Mannheim Heidelberg University Mannheim Germany; ^2^ Clinical Cooperation Unit Dermato‐Oncology German Cancer Research Center Heidelberg Germany; ^3^ Section of Clinical and Experimental Dermatology, Medical Faculty Mannheim Heidelberg University Mannheim Germany; ^4^ Institute of Pathology, University Medical Center and Medical Faculty Mannheim Heidelberg University Mannheim Germany; ^5^ Department of Dermatology, Lausanne University Hospital, Faculty of Biology and Medicine University of Lausanne Lausanne Switzerland; ^6^ Department of Dermatology, Venereology, and Allergology University Hospital Essen Essen Germany; ^7^ Department of Dermatology, Venereology, and Allergology University Hospital Würzburg Würzburg Germany; ^8^ Department of Dermatology, Venereology and Allergology University Medical Center Göttingen Göttingen Germany; ^9^ Section of Clinical and Molecular Dermatology, Medical Faculty Mannheim Heidelberg University Mannheim Germany; ^10^ European Center for Angioscience, Medical Faculty Mannheim Heidelberg University Mannheim Germany

**Keywords:** diffuse large B‐cell lymphoma, leg dermatoses, lymphoma, skin neoplasms, spontaneous neoplasm regression

## Abstract

Primary cutaneous diffuse large B‐cell lymphoma, leg type (PCDLBCL, LT) is a subtype of cutaneous B‐cell lymphoma with unfavorable prognosis usually requiring aggressive polychemotherapy for disease control. Only single cases of spontaneous regression of PCDLBCL, LT are reported in the literature, peaking 3 months post‐biopsy following a clinical history of no longer than 1 year. Here, we report the first case of a spontaneously relapsing and remitting PCDLBCL, LT with complete regression after a clinical history of more than 9 years and thus an atypically indolent clinical course. The female patient presented with recurrent erythematous, non‐ulcerated, non‐raised plaques of the right lower leg for 6 years. Pathological workup and exclusion of a systemic disease confirmed the diagnosis of PCDLBCL, LT. Due to the history of repeated spontaneous remission, no therapy was initiated. Nine years after first occurrence the patient presented with complete clinical remission lasting for 64 months. We retrospectively identified four additional PCDLBCL, LT patients with spontaneous remission lasting up to 53 months. Our data provide evidence for a distinct PCDLBCL, LT patient subgroup that clinicians should be aware of and warrants a watch‐and‐wait treatment regime.

## 1 INTRODUCTION

Primary cutaneous diffuse large B‐cell lymphoma, leg type (PCDLBCL, LT) is a subtype of cutaneous B‐cell lymphoma with an unfavorable prognosis and a 5‐year disease‐specific survival of about 50%–60%.^1^ Clinical presentation is commonly characterized by dark erythematous or livid, rapidly growing, ulcerative tumors, nodules or plaques usually located on one or both lower legs.^2^ Histologically, PCDLBCL, LT is marked by a diffuse dermal infiltrate primarily composed of a monotonous proliferation of centroblasts and/or immunoblasts with high mitotic activity.^1^, ^2^ The atypical lymphoid cells are usually immunoreactive for BCL2, MUM1, immunoglobulin M (IgM), and mostly BCL6.^1^, ^2^ Additionally, monoclonal rearrangement for immunoglobulin heavy (IgH) chains is present in most cases.^1^, ^2^ Nuclear factor kappaB‐activating mutations are observed frequently in *MYD88*, *PIM1*, and *CD79B*, of which *MYD88* ^L265P^ mutations are most common in about 75% of cases.^1^, ^3^ First‐line therapy includes R‐CHOP regimen polychemotherapy.^2^ Spontaneous regression of PCDLBCL, LT is very rare, with only six cases reported in the literature, occurring 3 months after biopsy at most, after a history not exceeding 1 year (Table 1, No. 6–11). Herein, we report the first case of a relapsing and remitting PCDLBCL, LT with an indolent course. It is characterized by a unique course of repeated spontaneous complete regression after multiple relapses over a period of more than 9 years. In addition, we describe the clinical, histological, and molecular characteristics of four additional cases retrospectively identified in collaboration with the German Dermatologic Cooperative Oncology Group (DeCOG) to identify common features and possibly a new subtype of PCDLBCL, LT.

**TABLE 1:** PDCLBCL, LT cases with spontaneous regression.

No.	Age/sex	Morphology	Location	History	Regression	Therapy	Remission duration	Last condition	Immuno‐histochemistry	Molecular analyses	IgH re‐arrangement	Ki‐67	EBER‐ISH	Inflammatory infiltrate
1^a^	53/F	Relapsing remitting plaques	Right leg	9 years	Independent of biopsy, recurrence elsewhere	None	64 months	Alive, stable remission	*Positive*: CD20, CD79a, BCL2, BCL6, MUM1, MYC, cIgM *Negative*: CD10, CD21	*MYD88* exon 5: wild type *BCL2* and *MYC* FISH normal *RAD51D* (p.L19F) and *NOTCH2* (p.N501K) mutations	Monoclonal	60%–80%	Negative	Diffuse, accentuated around B cell (peritumoral) T cell infiltrate (CD4:CD8 = 1:1, partially PD1 and FoxP3) but also intratumorally, dispersed
2	84/M	Nodules	Left leg	3 months	On administration of topical steroids and biopsy	None	32 months	Alive, stable remission	*Positive*: CD20, MUM1, BCL6	*MYD88* FISH normal	ND	ND	Negative	Sparse reactive T cell infiltrate (CD3, CD4:CD8 = 1:1)
3	83/M	Nodules	Both legs	9 months	1 month after biopsy	Prednisolone 5–20 mg daily and MTX 7,5 mg weekly (due to rheumatoid arthritis)	11 months	Dead (gastric carcinoma) 35 months after diagnosis	*Positive*: CD20, BCL2, MUM1 *Negative*: CD3, BCL6	*MYD88* mutation (point mutation c.794T>C; p.L265P); *PIM1* point mutation (c.268G>A; p.V90I)	ND	>90%	Negative	Sparse reactive T cell infiltrate
4	72/M	Nodule	Right arm	6 weeks	On excisional biopsy	Excisional biopsy	53 months	Alive, stable remission	*Positive*: CD20, CD79a, BCL2, MUM1 *Negative*: BCL6	*MYD88* FISH normal	Polyclonal	70%	Negative	Diffuse, accentuated around B cell (peritumoral) T cell infiltrate (CD4:CD8 = 1:1)
5	61/M	Nodule	Head, occipital left side	2 months	On excisional biopsy	Excisional biopsy	39 months	Alive, stable remission	*Positive*: CD20, BCL2, BCL6, MUM1	*MYD88* FISH normal	Monoclonal	60%	Negative	Diffuse, accentuated around B cell (peritumoral) T cell infiltrate (CD4:CD8 = 1:1) but also intratumorally, dispersed
6^4^	82/F	Nodules and plaques	Right leg	1 year	1 month after biopsy	None	4 months	Dead (stroke)	*Positive*: CD20, CD79a, BCL2, BCL6, MUM1 *Negative*: CD10, CD21, CD30	ND	Polyclonal	90%	Negative	Diffuse T cell infiltrate (CD3)
7^5^	72/F	Nodule	Left arm	1 month	On biopsy	Surgery	21 months	Alive, stable remission	*Positive*: CD20, CD79a, BCL2, MUM1 *Negative*: CD10	ND	ND	ND	Negative	Many T cells (CD3, CD4, CD8, perforin, granzyme B and TIA1)
8^6^	83/F	Nodules	Right leg	8 months	3 months after biopsy	RT	12 months	Alive, stable remission	*Positive*: CD20, CD79, BCL2, BCL6, MUM1, FoxP1, IgM *Negative*: CD5, CD10, CD21, CD30	ND	Monoclonal	>95%	Negative	Small‐sized T cells (CD2, CD3, CD8 predominating over CD4)
9^7^	66/M	Nodular plaque	Left leg	6 weeks	2 months after biopsy	RT	ND	Alive	*Positive*: CD20, CD79a, PAX5, BCL2, BCL6, MUM1 *Negative*: CD3, CD4, CD5, CD8, CD10, CD21, CD30, CD68, Cyclin D1, TdT	*MYD88* (point mutation c.794T>C)	Monoclonal	90%	Negative	Predominantly CD8^+^ T cell infiltrate
10^8^	79/M	Plaque	Left leg	1 week	1 month after biopsy	None	12 months	Recurrence after 1 year	*Positive*: CD20, MUM1, BCL2, BCL6	ND	ND	ND	ND	Minor component of T cells (CD3)
11^9^	62/M	Nodules	Left leg	1 month	3 months after biopsy	None	60 months	Alive, stable remission	*Positive*: CD20, CD79a, PAX5, MUM1, BCL2, BCL6 *Negative*: CD3, CD10, CD30, CD138	ND	Monoclonal	60%	Negative	ND

*Note*: No. 1–5: presented PDCLBCL, LT cases with spontaneous regression; No. 6–11: published PDCLBCL, LT cases with spontaneous regression.

Abbreviations: BCL2, apoptosis regulator BCL2; BCL6, B‐cell lymphoma 6 protein; CD3, T‐cell surface glycoprotein CD3; CD4, T‐cell surface glycoprotein CD4; CD5, T‐cell surface glycoprotein CD5; CD8, T‐cell surface glycoprotein CD8; CD10, Neprilysin; CD20, B‐lymphocyte antigen CD20; CD21, complement receptor type 2; CD30, tumor necrosis factor receptor superfamily member 8; CD68, Macrosialin; CD79a, B‐cell antigen receptor complex‐associated protein alpha chain; CD138, Syndecan‐1; FoxP1, Forkhead box protein P1; FoxP3, Forkhead box protein P3; IgM, immunoglobulin M; M, male; MTX, methotrexate; MUM1, PWWP domain‐containing protein MUM1; MYC, MYC proto‐oncogene protein; ND, not described; No, number; PAX5, paired box protein PAX5; PD1, programmed cell death protein 1; PCDLBCL, LT, primary cutaneous diffuse large B‐cell lymphoma, leg type; RT, radiotherapy; TdT, terminal deoxynucleotidyl transferase.

^a^ Unique case presented here with spontaneously relapsing and remitting course.

## 2 CASE PRESENTATION

**FIGURE 1:**
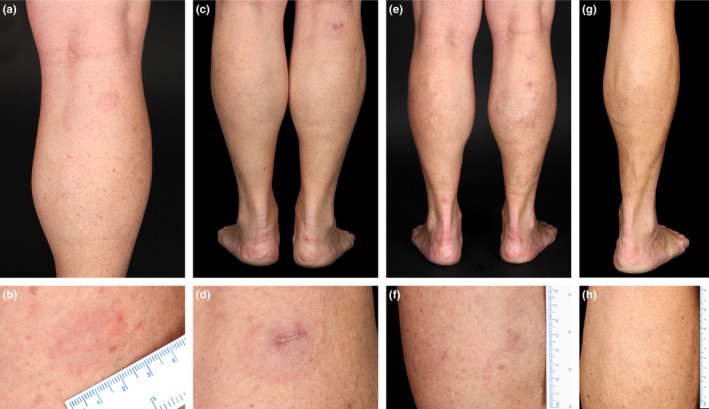
Clinical presentation. Annular erythematous plaque at the right calf (a, b) on initial presentation and (c, d) 2 months after biopsy. (e, f) Spontaneous regression and recurrence elsewhere at the right calf 1 year after initial presentation. (g, h) Spontaneous complete regression 3 years after initial presentation.

**FIGURE 2:**
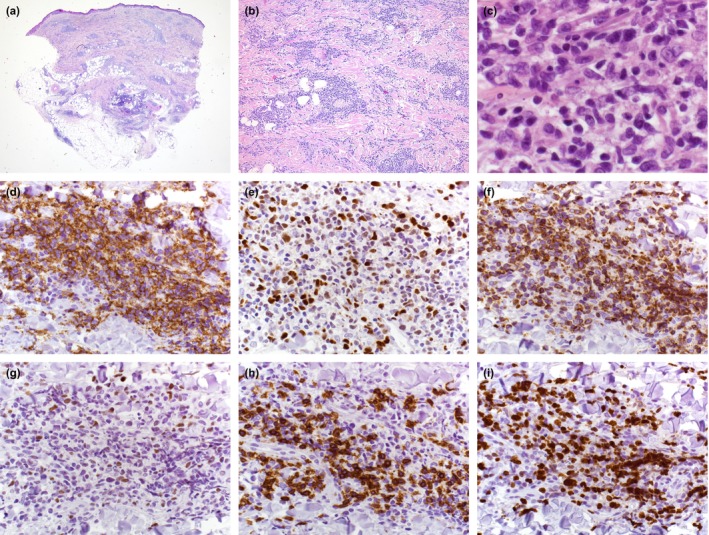
Histopathology of a punch biopsy. Nodular and interstitial infiltrate occupying the entire dermis and subcutis without epidermal involvement but with formation of a narrow grenz zone in the H&E stain (a‐c). The infiltrate consisted of large B cells, positive for (d) CD20, (e) MUM1 and (f) BCL2 and partly positive for (g) BCL6 in immunohistochemistry as well as small nests of (h) CD3‐positive T cells. High proliferative activity was detected by (i) Ki67 in the atypical lymphoid cells. Magniﬁcation: (a) x10; (b) x100; (c) x1000; (d‐i) x400.

A 53‐year‐old woman presented with relapsing and remitting skin alterations at the right lower leg for 6 years. The patient described an initial progression in the size of the lesion followed by spontaneous regression and recurrence limited to the right lower leg. Clinical examination yielded an annular, non‐ulcerated, erythematous flat plaque on her right calf (Figure 1a,b). Skin biopsy revealed nodular and diffuse lymphoid infiltration of the entire dermis and subcutis. No epidermal involvement, but formation of a narrow grenz zone were detected (Figure 2a). The infiltration consisted of medium‐ to large‐sized lymphocytes with large, round nuclei and numerous mitoses, consistent with sheets of centroblasts and immunoblasts (Figure 2b,c). In addition, an infiltrate of small lymphocytes and histocytes was found. The large lymphocytes stained positive for CD20, CD79a, BCL2, MUM1, MYC, and cytoplasmic IgM (cIgM), partly positive for BCL6 (Figure 2d–g) and negative for CD10 and CD21. The small lymphocytes were CD3‐positive (Figure 2h) and partially immunoreactive for CD4, CD8, PD1, and FoxP3, while CD68 identified the few scattered histiocytes. Proliferation marker protein Ki‐67 stained 60%–80% of the large cells (Figure 2i). *In situ* hybridization (ISH) showed no evidence of infection with Epstein–Barr virus (EBV) or translocation of *BCL2* or *MYC.* PCR identified monoclonal rearrangement of IgH. Mutation analysis revealed the *RAD51D* (p.L19F) and *NOTCH2* (p.N501K) mutations. However, *MYD88* exon 5 showed a wild‐type allele (Supporting Information Material and Methods). Blood analysis, bone marrow biopsy, lymph node ultrasonography, head magnetic resonance imaging, and neck, chest, abdomen, and pelvis computed tomography scans yielded no evidence of systemic disease. Thus, the diagnosis of PCDLBCL, LT was made. Due to her history of repeated spontaneous regressions, no therapy was initiated. Active surveillance in 3‐month intervals with clinical examination and repeated staging was performed (Table 1, No. 1 and Supporting Information Table S1). In the further course of the disease, the patient repeatedly developed non‐elevated, non‐ulcerated, erythematous plaques on the right lower leg followed by spontaneous regression (Figure 1c–f). Three years after the first presentation in our outpatient clinic, 9 years after first occurrence, complete regression of the skin alterations without recurrence elsewhere was noted (Figure 1g,h). In the follow‐up of 64 months no further skin alterations were detected (Supporting Information Table S1).

This exceptional clinical course prompted us to further screen within the German DeCOG for the presence of similar PCDLBCL, LT cases exhibiting spontaneous remission. Here, we identified four additional PCDLBCL, LT patients who showed spontaneous remission at different time points within the first year after disease onset (Table 1, No. 2–5). Only one patient experienced disease relapse after spontaneous remission. Diagnosis of PCDLBCL, LT was established by integration of histological morphology, immunohistochemistry, molecular pathology, and clinical examinations ruling out cutaneous manifestation of systemic lymphoma. Interestingly, all patients were male. The median age was 77.5 years. Location of the skin lesions included legs, arm, and occipital head. Remission occurred as early as 6 weeks after onset of the first skin manifestations and lasted for an observation period of up to 53 months without further lymphoma‐directed treatment. Three patients are still alive today without evidence for disease recurrence, while one patient died of gastric carcinoma. The immunophenotype was largely consistent in all four cases with positivity for B‐cell markers (CD20, CD79a) and expression of MUM1 and BCL2. However, BCL6 was negative in two patients. Molecular analyses revealed that one patient harbored both *MYD88* and *PIM1* mutations. Epstein–Barr encoding region ISH was consistently negative.

## 3 DISCUSSION

We here present a unique case of a spontaneously relapsing and remitting PCDLBCL, LT over a course of 9 years and most lately converted into complete remission for a 64‐month follow‐up period. To the best of our knowledge, this is the first report of a repeatedly occurring and remitting PCDLBCL, LT and the case with the longest disease history before development of spontaneous complete remission published hitherto. In addition to the exceptional course of the disease, this case exhibits a prominent reactive infiltration of T cells and histiocytes, which is presumably associated with spontaneous regression.

By presenting four additional PCDLBCL, LT cases with spontaneous regression, we substantiated the existence of this phenomenon in PCDLBCL, LT. Spontaneous regression of PCDLBCL, LT is believed to be very rare. Only six cases have been published (Table 1, No. 6–11). Excluding the presented case, in which the date of initial remission cannot be established unequivocally, spontaneous remissions occurred within the first year after disease, peaking after a disease course of 1–3 months.

Mechanistically, of the various mechanisms possibly involved in spontaneous regression, antitumoral immune infiltration is frequently discussed.^7^, ^10^ This is supported by the observation that high rates of spontaneous regression are found in immunotherapy‐responsive malignancies.^10^, ^11^ In fact, significant infiltrates of T cells and histiocytes were detected in several of the cases (Table 1), presumably contributing to spontaneous regression. As spontaneous remission in nearly all cases occurred in time‐related reference to the biopsy, it seems reasonable to suppose that the induced trauma is a critical factor in these patients and may have triggered the regression, probably by providing a stimulus for immune cell infiltration. However, this event alone seems insufficient to explain the distinct behavior and supposedly must coincide with specific features of the host immune system. Viral infections are well‐documented factor in self‐remission of malignancies.^12^ However, there was no evidence of infection, including EBV, in any of the cases presented (Table 1).

At the molecular level, we found no significant discriminative features with respect to the general PCDLBCL, LT population. However, in the relapsing and remitting case (Table 1, No. 1), we discovered mutations in two genes, *RAD51D* and *NOTCH2*, but not the prototypical *MYD88* ^L265P^ mutation. *RAD51D* is an established susceptibility gene in ovarian cancer, involved in DNA repair.^13^ *NOTCH2* mutations are frequently observed in diffuse large B‐cell lymphomas and occur with a frequency of about 5% in PCDLBCL, LT.^3^, ^14^ In another case, we detected a mutation in *PIM1* (Table 1, No. 3), which is postulated to represent a potential negative predictive factor for ibrutinib therapy in the activated B‐cell subtype of DLBCL.^3^, ^15^

In conclusion, we provide evidence for the possibility of self‐remission in PCDLBCL, LT. Spontaneous remission appears to be associated with a reactive infiltrate of T cells and histiocytes. Knowledge and further characterization of this extremely unusual course is critical for clinical management so prospective research should focus on risk stratification to meet the heterogeneity of this disease.

## CONFLICT OF INTEREST STATEMENT

None to declare.

## Supporting information


**Supporting Information.** Material and Methods
**Table S1.** Medical History Timeline
