# Supplementary material for: Spontaneous regression of primary cutaneous diffuse large B‐cell lymphoma, leg type: A case series and review of the literature
Source: J Dermatol. 2024 Jul 19;51(9):1233–9. doi: 10.1111/1346-8138.17339 (PMC11483968; doi:10.1111/1346-8138.17339)
Supplement: Supplementary file 1 — Supporting Information. Material and Methods Table S1. Medical History Timeline [file JDE-51--s001.pdf]

SUPPORTING INFORMATION

Spontaneous regression of primary cutaneous diffuse large B-cell lymphoma, leg type: a case series and review of the literature

Manuel Winkler[1,2,†], Jana Dorothea Albrecht[1,2,3,†], Christian Sauer[4], Theresa Kordaß[4], Emmanuella Guenova[5], Elisabeth Livingstone[6], Marion Wobser[7], Christina Mitteldorf[8], Cyrill Géraud[1,9,10,‡] and Jan Peter Nicolay[1,2,3,‡]

[1]Department of Dermatology, Venereology, and Allergology, University Medical Center and Medical Faculty Mannheim, Heidelberg University, Mannheim, Germany;

[2]Clinical Cooperation Unit Dermato-Oncology, German Cancer Research Center (DKFZ), Heidelberg, Germany

[3]Section of Clinical and Experimental Dermatology, Medical Faculty Mannheim, Heidelberg University, Mannheim, Germany;

[4]Institute of Pathology, University Medical Center and Medical Faculty Mannheim, Heidelberg University, Mannheim, Germany;

[5]Department of Dermatology, Lausanne University Hospital, Faculty of Biology and Medicine, University of Lausanne, Lausanne, Switzerland

[6]Department of Dermatology, Venereology, and Allergology, University Hospital Essen, Essen, Germany

[7]Department of Dermatology, Venereology, and Allergology, University Hospital Würzburg, Würzburg, Germany

[8]Department of Dermatology, Venereology and Allergology, University Medical Center Göttingen, Göttingen, Germany

[9]Section of Clinical and Molecular Dermatology, Medical Faculty Mannheim, Heidelberg University, Mannheim, Germany;

[10]European Center for Angioscience, Medical Faculty Mannheim, Heidelberg University, Mannheim, Germany.

† These authors share first authorship

‡ These authors share last authorship

The Journal of Dermatology

https://doi.org/10.1111/1346-8138.17339

Supporting Information Material and Methods

The patients provided informed consent to participate to this study. Photographs as well as punch biopsies containing epidermis, dermis and subcutis were taken with informed consent of the patients. Formaldehyde fixation, paraffin embedding, hematoxylin and eosin (H&E) stain and immunohistochemistry were carried out as per institutional standards. For analysis of the first case, the following primary antibodies were used: BCL2 (clone 124, M0887, Agilent Dako), BCL6 (clone PG-B6p, M7211, Agilent Dako), CD3 (clone LN10, MOB474, Diagnostic BioSystems, Pleasanton, CA, USA), CD20cy (clone L26, M0755, Agilent Dako, Agilent Technologies, Santa Clara, CA, USA), CD79a (clone JCB117, M7050, Agilent Dako), IgM (A0425, Agilent Dako), Ki67 (clone SP6, RBK027, Zytomed Sytems, Berlin, Germany), MUM1 (clone MUM1p, M7259, Agilent Dako), MYC (clone 9E10, 13-2500, Thermo Fisher Scientific, Waltham, MA, USA). Epstein-Barr encoding region (EBER) in situ hybridization (ISH) (Y5200, Agilent Dako), BCL2 ISH (Z-2192-200, Zytomed Sytems) and MYC ISH (Z-2090-200, Zytomed Sytems) were performed according to the manufacturer’s instructions. Polymerase chain reaction (PCR) using the BIOMED-2 primers was conducted to detect clonal immunoglobulin. For molecular pathological analysis a multi-biomarker next-generation sequencing (NGS) assay was carried out. On morphologically confirmed and enriched tumor tissue (tumor cell content >90%), 3781 DNA regions (amplicons) and 867 RNA amplicons were specifically amplified using the Oncomine Comprehensive Assay v3 (A35805, Thermo Fisher Scientific). NGS was performed using an Ion GeneStudio S5 system (Thermo Fisher Scientific) and mapped to the human genome assembly GRCh37 (hg19).

Supporting Information Table S1. Medical History Timeline

X – 2 years	First occurrence of relapsing remitting skin alterations at the right lower leg. Initially progression in size followed by spontaneous regression and recurrence elsewhere at the right calf.
Year X	Skin biopsy (external), including examination by an internationally recognized expert in cutaneous lymphomas: Initial diagnosis of primary cutaneous diffuse large B-cell lymphoma, leg type (PCDLBCL, LT). No evidence for lymph node or bone marrow involvement nor metastases. Spontaneous regression about 1 month after biopsy and recurrence elsewhere at the right calf.
X + 3 years	Skin biopsy (external): Aggressive NHL elsewhere was suspected, but not verified. PCDLBCL, LT appeared as less likely to the diagnosing pathologist on morphologic grounds, only. No evidence for lymph node or bone marrow involvement nor metastases.
X + 4 years	First presentation in our outpatient clinic. Relapsing remitting annular erythematous plaques at the right calf with spontaneous regression and recurrence elsewhere. Skin biopsy: PCDLBCL, LT. Hospitalization in our clinic for staging. No evidence for lymph node or bone marrow involvement nor metastases. Active surveillance and follow-up skin biopsy 4 months later: PCDLBCL, LT.
X + 5–6 years	Active surveillance in a 3-month interval with clinical examination, photographic documentation and repeated staging.
X + 7 years	Complete regression of the skin alterations without recurrence.
X + 8–13 years	Continued active surveillance without recurrence.
